# A Query Workflow Design to Perform Automatable Distributed Regression Analysis in Large Distributed Data Networks

**DOI:** 10.5334/egems.209

**Published:** 2018-05-25

**Authors:** Qoua L. Her, Jessica M. Malenfant, Sarah Malek, Yury Vilk, Jessica Young, Lingling Li, Jeffery Brown, Sengwee Toh

**Affiliations:** 1 Department of Population Medicine, Harvard Medical School and Harvard Pilgrim Health Care Institute, US

**Keywords:** Distributed regression, Distributed data networks, Privacy-protecting methods, Sentinel, Pharmacoepidemiology, PopMedNet™

## Abstract

**Introduction:**

Patient privacy and data security concerns often limit the feasibility of pooling patient-level data from multiple sources for analysis. Distributed data networks (DDNs) that employ privacy-protecting analytical methods, such as distributed regression analysis (DRA), can mitigate these concerns. However, DRA is not routinely implemented in large DDNs.

**Objective:**

We describe the design and implementation of a process framework and query workflow that allow automatable DRA in real-world DDNs that use PopMedNet™, an open-source distributed networking software platform.

**Methods:**

We surveyed and catalogued existing hardware and software configurations at all data partners in the Sentinel System, a PopMedNet-driven DDN. Key guiding principles for the design included minimal disruptions to the current PopMedNet query workflow and minimal modifications to data partners’ hardware configurations and software requirements.

**Results:**

We developed and implemented a three-step process framework and PopMedNet query workflow that enables automatable DRA: 1) assembling a de-identified patient-level dataset at each data partner, 2) distributing a DRA package to data partners for local iterative analysis, and 3) iteratively transferring intermediate files between data partners and analysis center. The DRA query workflow is agnostic to statistical software, accommodates different regression models, and allows different levels of user-specified automation.

**Discussion:**

The process framework can be generalized to and the query workflow can be adopted by other PopMedNet-based DDNs.

**Conclusion:**

DRA has great potential to change the paradigm of data analysis in DDNs. Successful implementation of DRA in Sentinel will facilitate adoption of the analytic approach in other DDNs.

## Introduction

Many research studies require pooling of patient-level information from multiple data sources to obtain sufficient sample size and more generalizable findings. Concerns about data security, patient privacy, unapproved use of data, and disclosure of proprietary information have limited these collaborations [1,2,3]. Data organized in a distributed data network (DDN) can mitigate these concerns [1,2,3]. Several DDNs already exist and have been used to investigate a wide range of clinical inquires in a distributed matter, including the Centers for Disease Control and Prevention’s Vaccine Safety Datalink [4], the National Institutes of Health (NIH)’s Health Care Systems Research Collaboratory [5], the U.S. Food and Drug Administration (FDA)’s Sentinel System [6], and the Patient-Centered Outcomes Research Institute (PCORI)’s National Patient-Centered Clinical Research Network (PCORnet) [7]. These networks allow data partners to retain physical control of their data while making multi-database analysis more secure and feasible [1,2,3].

Although simple descriptive and inferential analysis can be done with summary-level information (e.g., 2 × 2 tables of exposed and unexposed person-times and outcome events) in these networks, more complex statistical analysis has traditionally required sharing of patient-level information [8,9]. In recent years, researchers have developed and applied a number of newer analytic methods, including meta-analysis of site-specific effect estimates, methods that leverage confounder summary scores (e.g., propensity scores), and distributed regression analysis (DRA), to perform complex statistical analysis using only summary-level information [8,9,10,11,12,13,14,15].

**Figure 1:**
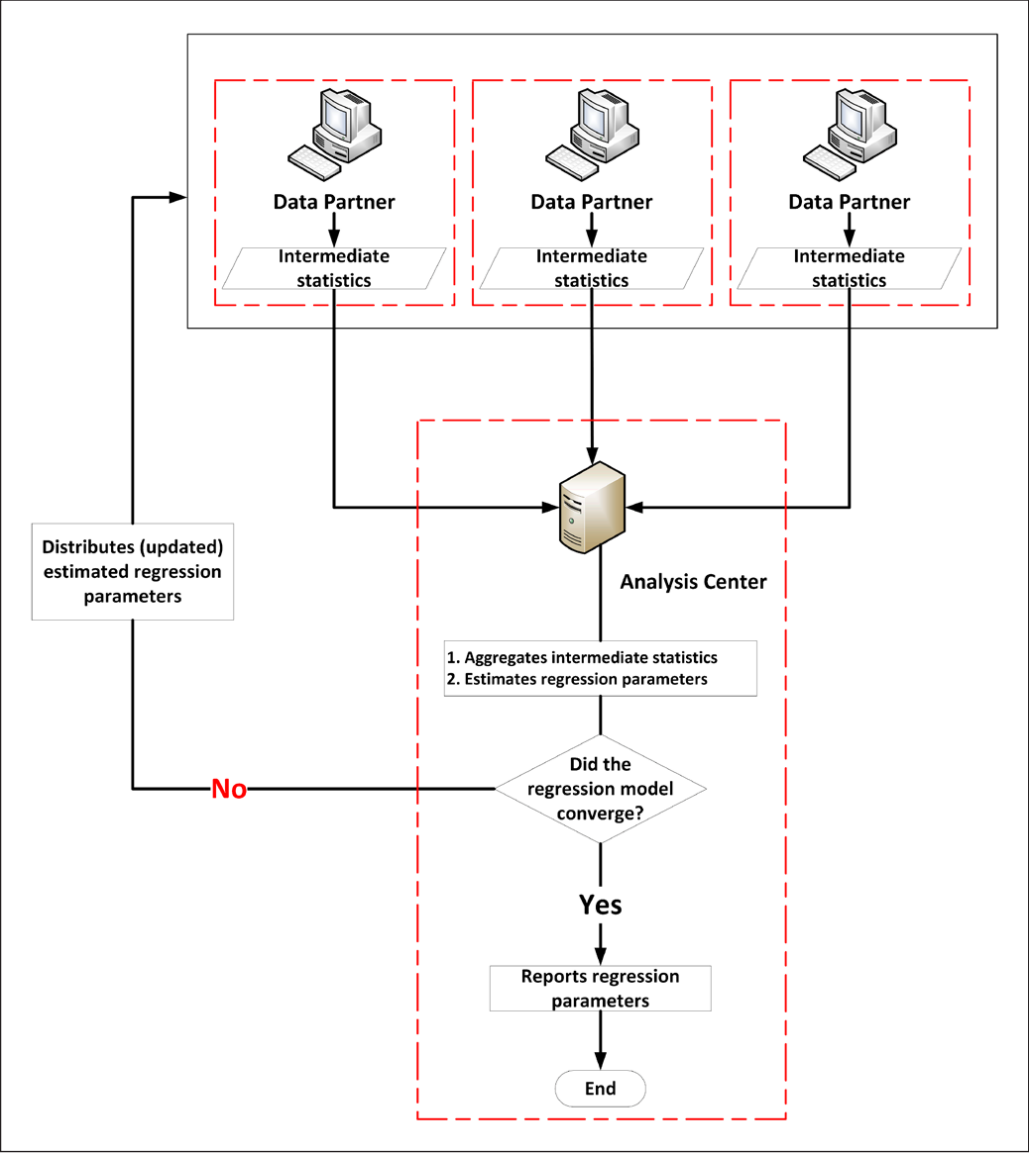
Iterative process to perform distributed regression analysis.

Many of these newer, more privacy-protecting analytic methods are promising. In particular, DRA requires only intermediate statistics (e.g., sums of squares and cross product matrix) to be shared, but produces statistically equivalent results as if the databases were pooled. This makes DRA a highly desirable analytic method within DDNs. Although researchers have successfully performed DRA in relatively small or simulated multi-database settings [12,13,14,15,16,17,18,19,20,21], routine implementation of the analytic method in practice is challenging. This is because convergence of some regression models common to biomedical research (e.g., logistic and Cox regression) is an iterative process that requires frequent exchanges of intermediate statistics among data partners and an analysis center (Figure 1). These iterations are resource-intensive and require extensive coordination. Routine use of DRA will require some automation of this process.

In this paper, we describe a DRA query workflow design and process framework for routine, large-scale, and automatable implementation of DRA using PopMedNet™. An open-source distributed networking software application, PopMedNet currently supports several large DDNs, including the Sentinel System, PCORnet, and the NIH Collaboratory [22].

## Methods

### Guiding principles for the design of query workflow and process framework to allow automatable distributed regression analysis

To maximize the applicability of the new DRA capability, our design sought to minimize disruptions to the established query workflow within PopMedNet and modifications to existing hardware configurations and software requirements of data sources that employ PopMedNet. The goal was to develop an automatable file transfer process that accommodates commonly used regression models (e.g., linear, logistic, and Cox) and allows users to specify different levels of workflow automation (completely manual, semi-automated, and fully automated).

### Study setting – The FDA Sentinel System

We developed the new DRA capability within the FDA-funded Sentinel System, one of the first DDNs that employed PopMedNet. The Sentinel System is a national surveillance system designed to monitor the safety of approved medical products using routinely collected electronic health data [6,23]. Sentinel has developed a suite of re-usable analytic tools and workflows to enable rapid identification of patient cohorts and comparative safety analyses in a DDN of 18 data partners. The Sentinel’s network architecture and tools have been adopted by other DDNs, including PCORnet and the NIH Collaboratory [22].

**Figure 2:**
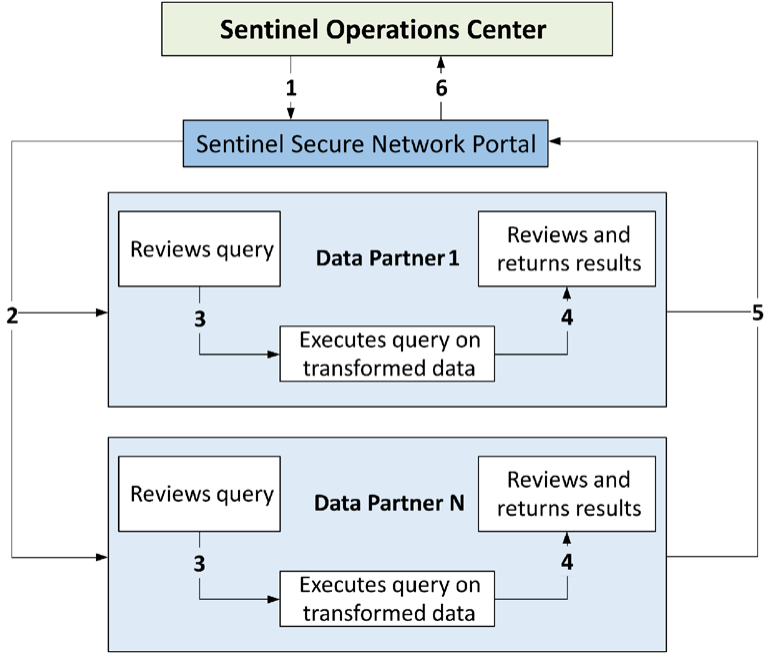
Query fulfillment process in the Sentinel System. Sentinel operations center (i.e., analysis center) creates and distributes query via the secure network portal supported by PopMedNet™.Data partners receive notification of the query and retrieve it from the secure network portal.Data partners review and execute query on their local, transformed data.Data partners review results.Data partners return results to the analysis center via the secure network portal.Sentinel operations center retrieves results from the secure network portal and performs final analysis. *Note:* Figure 2 is modified from Curtis et al. [24].

All Sentinel data partners transform their data into a common data model. The Sentinel operations center routinely checks the transformed data for completeness and consistency before using it for analysis. Sentinel has established a standard query fulfillment workflow for routine medical product safety assessment. The process begins with the FDA submitting a safety question to the operations center. A team comprised of FDA and Sentinel personnel defines query parameters such as exposures, outcomes, confounders, and inclusion and exclusion criteria based on established coding systems (e.g., International Classification of Diseases, Ninth or Tenth Revision, Clinical Modification, and National Drug Codes). Using the specifications, the operations center (which serves as the analysis center) assembles and tests a query package written in SAS (SAS Institute, Cary, NC). It then securely distributes the final package to each data partner through PopMedNet for local execution on the transformed data (Figure 2). Data partners produce and securely transfer the requested information, usually in aggregated form, back to the operations center for final analysis through PopMedNet. Detailed patient-level data remains behind the data partners’ firewalls, protecting patient privacy and proprietary information. Detailed description of the Sentinel query process is available elsewhere [24].

Sentinel’s analytic capabilities largely revolve around its ability to rapidly identify cohorts of interest with its validated, customizable Cohort Identification and Descriptive Analysis (CIDA) tool [25]. This tool includes a set of SAS programs that contain editable macro parameters and input files to define query parameters. It offers considerable query customization and analytic flexibility. The tool also can create a de-identified patient-level analytic dataset to be stored locally at each data partner site. The dataset can then serve as an input file for other re-usable Sentinel tools (e.g., propensity score analysis tool, and the DRA algorithm) for inferential analysis [26,27].

A typical Sentinel query involves four network folders (*sasprogram, inputfiles, dplocal*, and *msoc*) at each data partner, collectively known as the common folder structure. The *sasprogram* and *inputfiles* folders contain the necessary files required for local execution of the analysis on the data partner’s transformed data. More specifically, the *sasprogram* folder contains the SAS programs and macros while the *inputfiles* folder includes lookup tables, codes, or files used to define the covariates or other parameters of the analysis. The *dplocal* folder houses the de-identified patient-level dataset generated upon successful execution of the CIDA package; this dataset remains behind the data partner’s firewall. The *msoc* folder stores the output file(s) or dataset(s), typically summary-level, requested by the query; they are the only files that are transferred to the operations center.

### PopMedNet

PopMedNet (https://www.popmednet.org) has served as the Sentinel data-sharing platform since 2011. Two interfaces interlink the network topology of PopMedNet: a web-based network portal and the DataMart Client (DMC). The web-based portal is typically used by the analysis center (e.g., the Sentinel operations center) to create, distribute, and manage queries. The DMC is a locally installed Microsoft Windows® application that acts as an inbox for data partners to receive query packages and transfer results to the analysis center. All file transfers (query requests and responses) between the data partners and the analysis center are achieved through secure HTTPS/SSL/TLS connections. There are no virtual machines, open ports, Virtual Private Networks, or any external access to data partner data, abating concerns about data security and unauthorized access [28,29]. The system ensures only approved queries are submitted to and responses returned by participating data partners with several levels of software-enabled governance [29]. The PopMedNet web portal and related Sentinel Systems are hosted in a Federal Information Security Management Act (FISMA)-compliant data center. Third-party code audits and penetration tests are conducted on PopMedNet infrastructure annually [30].

### Data partner technological configurations

As part of the development process, we surveyed all Sentinel data partners to catalogue their hardware and software configurations to help guide our DRA query workflow design. Sixteen of the 18 participating data partners responded to the survey. There are currently three general configurations of the components (DMC, SAS, and the common folder structure) required to fulfill a query. In five data partners, these components are available on the same Windows® desktop computer or server (Configuration 1). Three data partners house all components on different Windows® machines (Configuration 2) and eight have the components installed on different machines with different operating systems (e.g., the DMC on a Windows® desktop computer, while SAS and the common folder structure on a Linux server) (Configuration 3). These configurations dictate data partners’ DMC access to the contents of the common folder structure (e.g., CIDA output). The DMCs at four data partners have direct access to these contents, while 11 implemented a manual process of transferring the common folder structure to the DMC computer or an accessible drive. Although we could not obtain information from some of the data partners, we expect them to fall under one of the three configurations identified.

## Results

### A three-step process framework to allow automatable distributed regression analysis in PopMedNet

**Figure 3:**
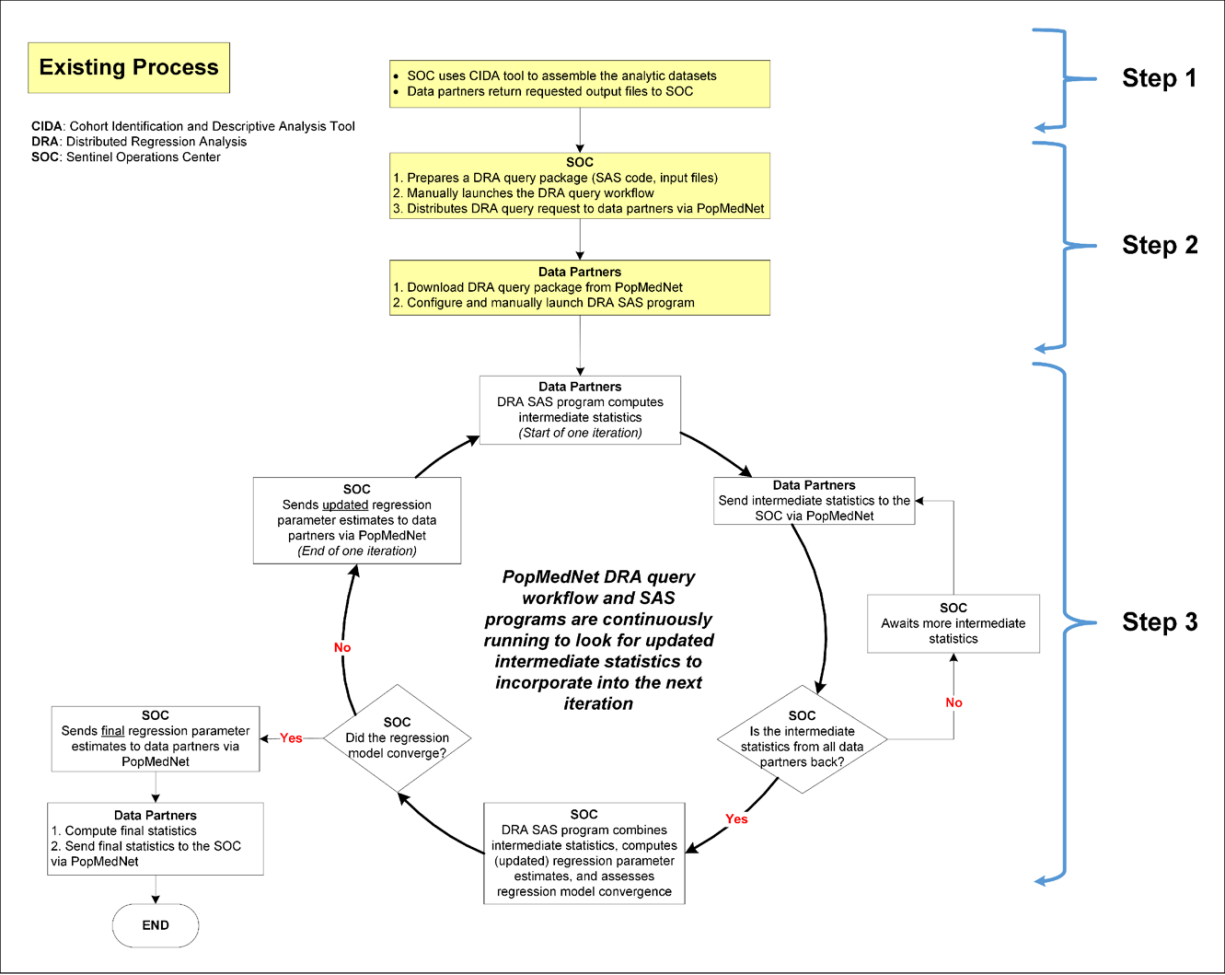
A 3-step process to conduct automatable distributed regression analysis within PopMedNet™.

We have developed and implemented a three-step process framework for routine DRA in large DDNs that employed PopMedNet as their distributed data-sharing platform:

1. Assemble a de-identified patient-level analytic dataset at each data partner site using a distributed program developed by the analysis center.
2. Distribute a DRA package to each data partner for local iterative analysis through PopMedNet.
3. Iteratively transfer intermediate files between data partners and the analysis center until the regression model converges or until the analysis reaches a pre-specified maximum number of iterations (Figure 3).

The process framework can leverage the existing query fulfillment process in Sentinel and query workflow in PopMedNet to complete Steps 1 and 2; Step 3 required enhancements to the existing PopMedNet infrastructure.

#### Step 1: Assemble an analytic dataset at each data partner site

In the first step, the analysis center distributes a CIDA package via PopMedNet to assemble a de-identified patient-level analytic dataset at each data partner site (Figure 3: Step 1). The patient-level dataset includes eligible patients and covariates of interest, as specified by the requester. Consistent with the existing Sentinel query fulfillment process, this dataset is stored in the *dplocal* folder and not transferred to the analysis center. This step can also accommodate additional *ad hoc* SAS code to modify or add covariates that are not part of standard CIDA output.

#### Step 2: Distribute a distributed regression analysis query package to data partners for local iterative execution

In the second step, the analysis center distributes a DRA package to all participating data partners via PopMedNet (Figure 3: Step 2). This package utilizes the common folder structure to organize the required analytic components for DRA. The *sasprogram* folder includes a main DRA SAS program and the *inputfiles* folder contains initial and subsequent iterative “guesses” of the regression parameter estimates and the required DRA macros. Upon receiving the package, data partners unzip the package, manually edit the main SAS program to specify the location of the unzipped DRA package, and execute the main SAS program on the de-identified patient-level dataset created in Step 1. This main program is set to run continuously.

#### Step 3: Iteratively transfer files between data partners and the analysis center

Successful execution of the main DRA SAS program outputs an intermediate statistics file to the *msoc* folder (Figure 3: Step 3). Data partners then upload and transfer the output file to the analysis center via PopMedNet. A corresponding SAS program at the analysis center also runs continuously to accept and aggregate the intermediate statistics from all participating data partners, update the regression parameter estimates, and evaluate model convergence. If the model convergence criteria are not met, the updated parameter estimates are re-distributed to the data partners via PopMedNet and used as new “guesses” of the regression parameter estimates. This process of local iterative execution and transferring files between the data partners and analysis center continues until the model converges or a pre-specified maximum number of iterations has been reached.

### Enhancements to PopMedNet infrastructure to allow automatable distributed regression analysis

From the perspective of the PopMedNet query workflow, we can view DRA as a single query request that contains multiple sub-query requests and responses (iterations) looking for the “converging” intermediate statistics. The current query workflow used in production manually supports one sub-query request and response (Figure 4a). Manual transfer of files over multiple iterations will be too resource-intensive and restrict the practicability of DRA in DDNs. Therefore, we enhanced the PopMedNet query workflow to allow automatable iterative transfer of files between data partners and the analysis center (Figure 4b).

**Figure 4a:**
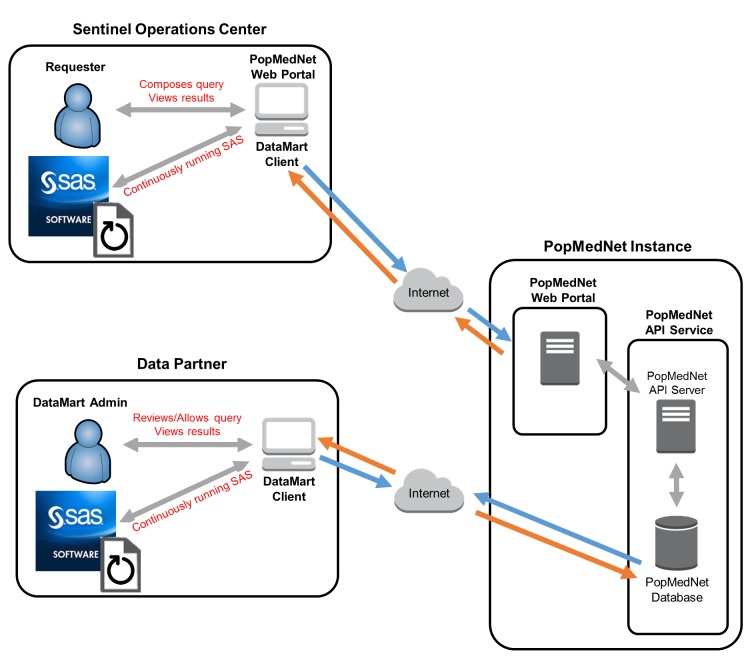
Current PopMedNet™ query workflow in production.

**Figure 4b:**
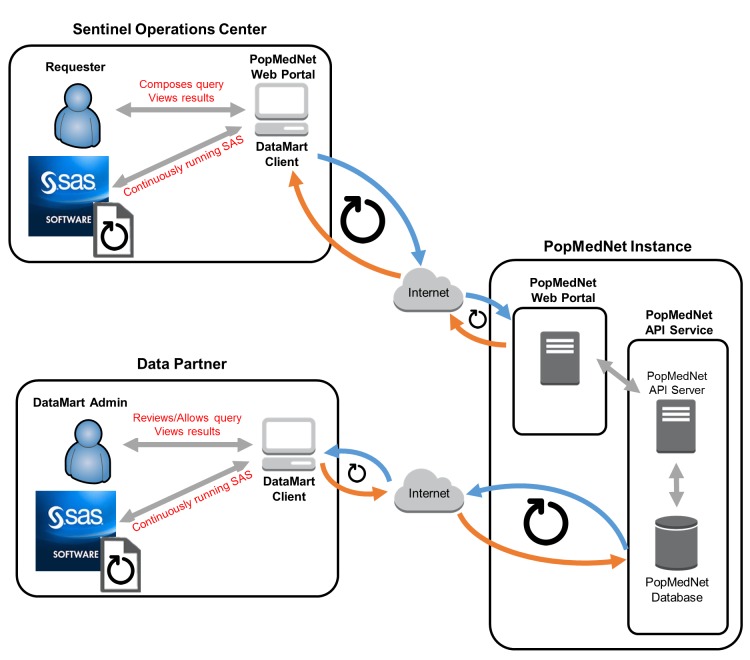
Enhanced PopMedNet™ query workflow to support automatable distributed regression analysis. *API:* application programming interface.

To achieve this, we built a new back-end component referred to as a “DRA adapter” in PopMedNet. This adapter allows the data partners and analysis center to have the option to automatically upload files from and download files to pre-defined folders, when a specific trigger text file appears. To trigger these automatable processes, we also built into the adapter a DMC functionality that monitors pre-defined folders for the appearance of trigger text files. This functionality is initiated at the start of Step 2 of the process framework and continues until the model converges or the analysis reaches a pre-specified maximum number of iterations. In addition, DRA requires iterative distribution of updated regression parameter estimates from the analysis center to the data partners. The current workflow used in production only allows one set of input files per query request. We enhanced this functionality to associate files to each sub-query request, allowing multiple sets of input files and response files to be associated to one DRA query request.

**Figure 5:**
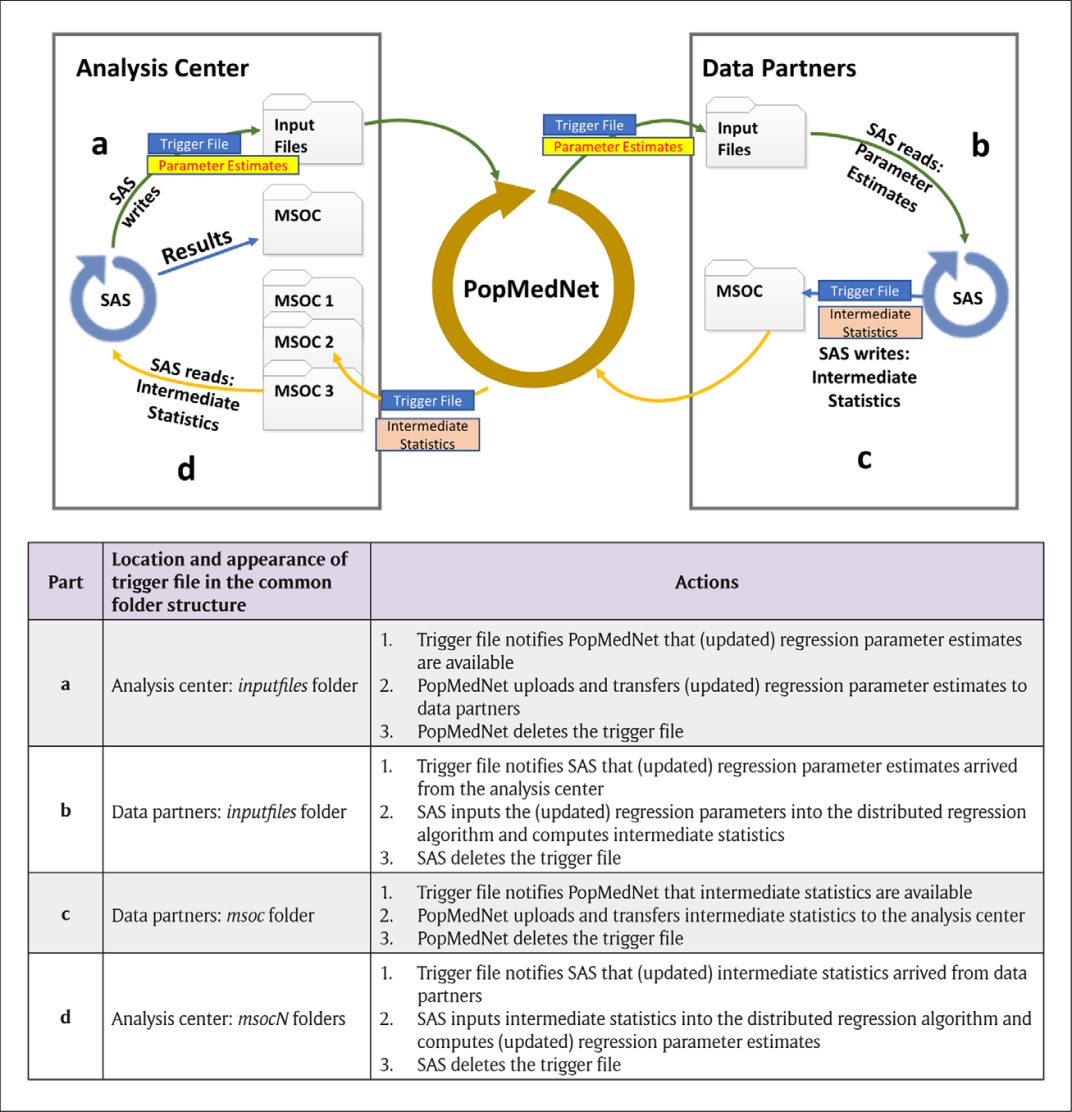
Trigger file and actions to allow automated distributed regression analysis in PopMedNet™.

We integrated the new automatable iterative file transfer process—made possible by the new adapter—with the SAS-driven DRA analytic process to allow Step 3 of the framework described in Figure 3. The integration leverages the existing Sentinel common folder structure and uses trigger text files and the newly developed DMC folder monitoring functionality to iteratively and sequentially initiate one process after the other (Figure 5). At the beginning of each iteration, the analysis center distributes initial or new “guesses” of the regression parameter estimates to the *inputfiles* folder of each data partner via PopMedNet (Figure 5: Parts a and b). PopMedNet then creates a trigger text file (*files_done.ok*) to signal to the continuously running DRA SAS program at each data partner site to incorporate the new guesses into their local execution of the program on the de-identified patient-level dataset. The DRA program outputs intermediate statistics to the *msoc* folder along with a trigger text file (*files_done.ok*) (Figure 5: Part c). This trigger file signals to PopMedNet that intermediate statistics are computed and are ready to be uploaded for transfer to the analysis center. Upon completion of the upload of the files, PopMedNet deletes the trigger file from the *msoc* folder. This step ensures that the appearance of a new trigger file in the next iteration will automatically initiate a new file transfer process.

PopMedNet then transfers each data partners’ files to their designated folder at the analysis center (Figure 5: Part d). Upon completion of the transfer, PopMedNet creates and deposits a trigger text file into each of the data partner-designated folders (e.g., *msoc1, msoc2*, etc). The appearance of the trigger text files prompts the SAS program at the analysis center to: 1) assess model convergence using the returned intermediate statistics, 2) output updated regression parameter estimates to the *inputfiles* folder, and 3) delete the trigger text files that initiated the computation process at the analysis center. Again, the last step ensures the appearance of new trigger files will automatically initiate a new computation process at the analysis center in the next iteration. If necessary, the new “guesses” are re-distributed to the data partners using the file transfer process described above.

This process of transferring files and computing statistics at the data partners and analysis center continues iteratively until the regression model converges or it reaches a pre-specified maximum number of iterations. If either of these two conditions is met, the SAS program at the analysis center outputs a different trigger text file (*job_done.ok*) to the *inputfiles* folder, and PopMedNet transfers this file to the data partners to invoke SAS code to compute diagnostic statistics (e.g., goodness-of-fit test, area under the receiver operating characteristics curves). These statistics are returned to the analysis center in the same manner as described above and all SAS programs and the folder monitoring functionality of DMCs are terminated.

## Discussion

We have developed a new query workflow to conduct automatable DRA using PopMedNet. We have also developed and validated SAS programs that perform distributed linear, logistic, and Cox proportional hazards regression analysis, which we will describe in future publications. The developed workflow requires minimal modifications to existing configurations at data sources that employ PopMedNet, is agnostic to statistical software (e.g., SAS and R), accommodates different regression models (e.g., linear, logistic, and Cox), and allows different levels of automation of the iterative file transfer process between data partners and the analysis center (completely manual, semi-automated, and fully automated). The DRA process framework involves three steps:

1. Assembling a de-identified patient-level analytic dataset at each data partner using a distributed program developed by the analysis center.
2. Distributing a DRA package developed by the analysis center for iterative local execution at each data partner.
3. Iteratively transferring intermediate statistics between data partners and the analysis center until the regression model converges or a pre-specified maximum number of iterations has been reached.

We developed this new capability within the Sentinel System. The existing query fulfillment process in Sentinel allows us to create the analytic dataset (Step 1) and distribute the DRA package (Step 2) without modifications to the process. We enhanced the PopMedNet query workflow to support iterative, automatable file transfers between data partners and the analysis center in the form of sub-query requests and responses embedded within an overall DRA query request. Trigger text files at different steps of the workflow integrate and automate the PopMedNet-driven file transfer process and the SAS-driven analytic process.

### Automatable distributed regression analysis and its acceptability in distributed data networks

To our knowledge, DRA has not been implemented or used routinely in large DDNs. This is likely attributed to the resources required to integrate technology, appropriate governance, and user acceptance. Although we have developed the technical capability to conduct fully automatable DRA, the degree of automation will depend on user acceptability. In addition to collecting information on data partners’ technology configurations to inform our development work, we also inquired about their perspectives towards automating part or all of the query workflow, such as automatic file uploads and downloads. The reactions were mixed. Six data partners would be willing to automate these steps, one would require approval from their technical governing board, and eight would not be willing to automate any of these steps. Most data partners require or prefer the option to review all files prior to upload or download.

The developed DRA capability allows users to set the workflow to be manual, but this will likely impede routine use of DRA and data partner participation due to its tediousness and susceptibility to human errors. However, a manual DRA workflow is likely required as part of the initial roll-out phase to build trust and acceptability from data partners. Having an opportunity to review and confirm that the iterative process only transfers highly summarized, non-identifiable intermediate statistics may improve data partners’ willingness to automate some or all of these steps.

### Comparison with prior work

There have been previous efforts in making DRA a more practical analytic option in DDNs, including the SCAlable National Network for Effectiveness Research (SCANNER) and WebGLORE [19,20,21]. Our three-step process framework is similar to those proposed by SCANNER and WebGLORE. WebGLORE identified four modules (steps) that are required for their DRA workflow: 1) user registration, 2) initiator task creation, 3) user participation, and 4) collaborative model construction. SCANNER partitioned DRA into nine steps that included additional functionalities of assigning staff roles with different levels of authorization and allowing approval of actions or results at different points of the workflow. Step 1 in WebGLORE is generally not required in established DDNs like Sentinel as all data partners are known to the analysis center, steps 2 and 3 are embedded in Step 2 of our process framework, and step 4 is synonymous with Step 3 of our framework. Similarly, steps 1 to 4 in SCANNER are embedded in Steps 1 and 2 of our process framework, and steps 5 to 9 is synonymous with Step 3 of our framework.

Notably, the development of the SCANNER software, which allows web-based DRA in DDNs, was informed in part by PopMedNet. However, there are several key differences between SCANNER and the approach described in this article. Specifically, DDNs must install a virtual machine and open appropriate ports to the master node hosting the SCANNER hub to implement DRA. Based on the feedback from the Sentinel data partners and our experience in several DDNs, these requirements would likely pose substantial challenges to the adoption of the software. Indeed, data partners unwillingness to install new software, security concerns with unauthorized access, and platform incompatibility were previously identified as potential challenges to adopting WebGLORE [20].

On the other hand, our design requires no new software installation or major modifications to the existing configurations in data sources that employ PopMedNet. There are no virtual machines, open ports, Virtual Private Networks, or external access to the data at the data partners or the analysis center. All file transfers and communications are done through PopMedNet, which has undergone several third-party software security assessments and code reviews [29,30]. Both DMC and SAS are run under the user accounts that the data partners create and maintain. Heterogeneity in technological configurations across Sentinel data partners does exist, but will likely not be an issue with our workflow, which is designed to accommodate existing configurations.

### Extension of the current work to other distributed data networks

Although we chose to develop this new PopMedNet capability within the Sentinel System, the DRA components were developed and implemented in the core PopMedNet code base and can be leveraged by other PopMedNet-based DDNs, such as PCORnet and the NIH Collaboratory. Most PopMedNet-based DDNs require the same components as Sentinel to fulfill a query: a DMC at each data partner to receive and respond to query requests, a common folder structure to manage and organize query results, and SAS to perform statistical analysis. Most of the data partners in other DDNs will likely have one of the three major configurations identified among the Sentinel data partners. Thus, the new DRA capability can be extended to other PopMedNet-based DDNs. Importantly, data partners that are participating in our ongoing beta-testing are also members of other DDNs, such as PCORnet, thus successful testing and implementation of the DRA query workflow within these data partners will facilitate adoption of automatable DRA in other DDNs.

### Future work

To our knowledge, there is no published experience on addressing data partners’ computer system failures or interruptions between DRA iterations. The current Sentinel query workflow addresses interruptions by re-running the query. This strategy is not optimal for automatable DRA, as all data partners would have to re-initiate the whole DRA workflow starting in Step 3. Future enhancements will include the capability to pause the process or restart the analysis from the previous error-free iteration when an interruption occurs. Although the automatable file transfer process is agnostic to regression models and statistical software, additional work is needed to develop analytic code for other more complicated statistical models and statistical software.

We have developed the DRA capability within horizontally partitioned data environments, a setting in which information from different individuals is available in different data sources. DRA can also be applied to vertically partitioned data environments [31,32,33], a setting in which information from the same individual is recorded in multiple data sources. DRA of vertically partitioned data will require development of new analytic code, but the PopMedNet-driven automatable file transfer process can be used in this setting with minimal modifications. Lastly, we have developed a workflow with three different levels of automation and the ability to review files prior to download, upload, and transmission to facilitate data partner acceptability of DRA. Future work would be to enhance our workflow to include other secure multiparty computation protocols with these functionalities [16,34].

## Conclusion

We have developed a new query workflow to perform automatable DRA in PopMedNet-driven DDNs. The workflow is currently being piloted in the Sentinel System, a national medical product safety surveillance system. Components of this workflow and the process framework are likely extendable to other DDNs. Successful implementation of DRA functionality in Sentinel will likely lead to adoption of DRA in other DDNs.
